# Characteristic comparison of triglyceride-rich remnant lipoprotein measurement between a new homogenous assay (RemL-C) and a conventional immunoseparation method (RLP-C)

**DOI:** 10.1186/1476-511X-7-18

**Published:** 2008-05-17

**Authors:** Hiroshi Yoshida, Hideo Kurosawa, Yuji Hirowatari, Yutaka Ogura, Katsunori Ikewaki, Ikuro Abe, Shinichi Saikawa, Kenichi Domitsu, Kumie Ito, Hidekatsu Yanai, Norio Tada

**Affiliations:** 1 Department of Laboratory Medicine, Jikei University Kashiwa Hospital, Japan; 2 Department of Internal Medicine, Jikei University Kashiwa Hospital, Japan; 3 Department of Clinical Laboratory, Jikei University Hospital, Japan; 4 Bioscience Division, TOSOH Corporation, Japan; 5 Division of Cardiology, Jikei University School of Medicine, Japan

## Abstract

**Background:**

Increased serum remnant lipoproteins are supposed to predict cardiovascular disease in addition to increased LDL. A new homogenous assay for remnant lipoprotein-cholesterol (RemL-C) has been developed as an alternative to remnant-like particle-cholesterol (RLP-C), an immunoseparation assay, widely used for the measurement of remnant lipoprotein cholesterol.

**Methods:**

We evaluated the correlations and data validation between the 2 assays in 83 subjects (49 men and 34 women) without diabetes, hypertension and medications for hyperlipidemia, diabetes, and hypertension, and investigated the characteristics of remnant lipoproteins obtained by the two methods (RLP-C and RemL-C) and their relationships with IDL-cholesterol determined by our developed HPLC method.

**Results:**

A positive correlation was significantly found between the two methods (r = 0.853, 95%CI 0.781–0.903, p < 0.0001). Bland & Altman analysis revealed that RemL-C values were likely to be significantly higher than RLP-C values, particularly in samples with high levels of remnant lipoproteins. Several data dissociations between the RemL-C and RLP-C were also observed. The HPLC chromatograms show high concentrations of chylomicron cholesterol in serum samples with RemL-C level < RLP-C level, but high concentrations of IDL-cholesterol in samples with RemL-C level > RLP-C level. RemL-C (r = 0.339, 95%CI 0.152–0.903; p = 0.0005) significantly correlated with IDL-cholesterol, but not RLP-C (r = 0.17, 95%CI -0.047–0.372; p = 0.1237) in all the samples (n = 83).

**Conclusion:**

These results suggest that there is generally a significant correlation between RemL-C and RLP-C. However, RemL-C assay is likely to reflect IDL more closely than RLP-C.

## Background

Hypertriglyceridemia is a heterogeneous disorder of lipoprotein metabolism with a less definite association to atherosclerosis risk than hypercholesterolemia or increased low-density lipoprotein (LDL)-cholesterol [1]. Patients with moderate hypertriglyceridemia such as familial combined hyperlipidemia, diabetic dyslipidemia, or metabolic syndrome more often develop premature atherosclerotic diseases, because smaller-sized triglyceride (TG)-rich lipoproteins such as chylomicron remnants and very-low-density lipoprotein (VLDL) remnants penetrate the arterial intima from plasma, than larger-sized chylomicrons [1-6]. Remnant lipoproteins are atherogenic, and elevated remnant lipoproteins are associated with the increased risk of cardiovascular disease [2-8].

Two clinically available methods to determine cholesterol levels of remnant lipoproteins have ever been developed, but these assay procedures are basically different. First, remnant-like particle-cholesterol (RLP-C), an immunoaffinity separation method (RLP-C assay; Otsuka, Japan) was developed, and this assay isolates remnant-like particles (RLPs) from human serum using an immunoaffinity gel containing two different immobilized monoclonal antibodies to human apolipoproteins A-1 and B-100 [9,10]. Many clinical studies have demonstrated that RLP-C is a risk factor for cardiovascular disease, and serum RLP-C levels are higher in patients with coronary artery disease, diabetes, and metabolic syndrome than in healthy subjects [2,4,11]. Thus, RLP-C measurement can be performed without an ultracentrifugation, but it takes some time and is not able to be run on an autoanalyzer.

Next, Remnant Lipoprotein Cholesterol Homogenous assay (RemL-C assay; Kyowa Medex, Japan) was developed, and this assay utilizes special surfactant [polyoxyethylene-polyoxybutylene (POE-POB) block copolymer] and phospholipase D, which can selectively solubilize and degrade TG-rich remnant lipoproteins, VLDL remnants and chylomicron remnants [12,13]. In contrast to RLP-C assay, RemL-C assay is able to be performable on a universal autoanalyzer, thereby allowing quick and high throughput measurements. Nakada et al [13] reported that remnant lipoproteins, measured by RemL-C, were increased in patients with coronary artery disease, indicating the clinical significance of coronary risk assessment by remnant lipoprotein levels measured by RemL-C. In samples from patients with diabetes, RemL-C correlated with RLP-C, but discrepant data between the 2 methods were found investigated by the gel filtration method, suggesting a murky difference in the affinity of respective assay reagents to various TG-rich lipoproteins [12].

We developed a novel high performance liquid chromatography (HPLC) method for measuring cholesterol levels in the major classes of serum lipoproteins within 25 min using an anion exchange column filled with diethylaminoethyl-ligand nonporous polymer-based gel by elution with a step gradient of sodium perchlorate concentration [14,15]. This HPLC method is able to determine cholesterol levels of HDL, LDL, IDL (intermediate-density lipoprotein), VLDL, and chylomicron similarly to the ultracentrifugation method, a golden standard method to determine cholesterol levels of lipoprotein fractions despite its the technical complexity inappropriate for routine clinical laboratory use. Cholesterol values of HDL, LDL, IDL, VLDL and chylomicron measured by this HPLC method are correlated to those estimated by the ultracentrifugation method [14], and therefore this HPLC method may be employed as a substitute of the ultracentrifugation method. RLP-C correlated well with VLDL-cholesterol but poorly with IDL-cholesterol, measured by the HPLC method [14] similarly as RLP-C was correlated to VLDL-cholesterol but not to IDL-cholesterol, measured by the ultracentrifugation method [10]. However, the associations of RemL-C with VLDL and IDL have never been examined quantitatively by the HPLC method, although the RemL-C values contain VLDL remnant (IDL) cholesterol concentrations qualitatively estimated by the gel filtration method and polyacrylamide gel electrophoresis analysis in the previous report [12].

In the context, we evaluated the correlations and data validation between the 2 assays (RLP-C and RemL-C) in subjects without diabetes, hypertension and medications for hyperlipidemia, diabetes, and hypertension, and investigated the characteristics of remnant lipoproteins obtained by the two assays and their relationships with IDL-cholesterol determined quantitatively by our HPLC method.

## Methods

### Specimens

We tested 83 clinical samples of fasting sera consecutively obtained from patients (49 men and 34 women), aged 61 ± 10 years, without diabetes, hypertension and medications for hyperlipidemia, diabetes, and hypertension at outpatient clinics of Jikei University Hospital, Japan. Fasting samples were used in the present study because dietary fats may influence postprandial lipoprotein status, but fasting states in the study subjects were not necessarily completed for more than 12 hours. All subjects gave informed consent to participate in the present study. This study protocols were approved by the ethics committee of Jikei University School of Medicine. Blood samples were allowed to clot at room temperature and immediately were centrifuged at 2000 × g for 15 minutes to prepare serum samples. All serum samples were analyzed immediately after serum preparation.

### Determination of cholesterol concentrations of remnant lipoproteins

Cholesterol concentrations of remnant lipoproteins were measured by the RLP-C assay (Otsuka, Japan) and the RemL-C assay (Kyowa Medex, Japan).

RLP-C assay isolates remnant-like particles (RLPs) from human serum using an immunoaffinity gel containing two different immobilized monoclonal antibodies to human apolipoprotein A-1 (H-12) and B-100 (JI-H) [9,10]. RLP-C assay was performed according to manufacturer's procedures. Briefly, a-3 mm diameter stainless steel bead (Otsuka, Electronics) was added to each micro sample cup (Boehringer Mannheim); 300 μl of the immunoaffinity gel and 5 μl of specimen were then added to each cup, and the mixture was incubated at room temperature on a mixer with build-in magnetic bars to sufficiently mix by driving the beads up and down in each cup. After 2 hour, the supernatant (unbound fraction) was transferred for cholesterol quantification by an enzymatic assay. The cholesterol values were multiplied 61-fold to reflect dilution of the specimen at the immunoseparation step.

RemL-C assay utilizes surfactant POE-POB block copolymer and phospholipase D, which can selectively solubilize and degrade TG-rich remnant lipoproteins, VLDL remnants and chylomicron remnants [12,13]. Then, released cholesterol was measured enzymatically. POE-POB selectively binds to VLDL remnants and IDL particles, and the phospholipase D addition supports the reactivity towards chylomicron remnants. This RemL-C assay was performed with a universal autoanalyzer (Hitachi 7600 automated analyzer, Japan).

### Determination of IDL cholesterol and lipoprotein profile by the HPLC method

Cholesterol levels of fractionated lipoproteins were measured by our developed HPLC method [14,15]. Briefly, serum lipoproteins were separated on a column containing diethylaminoethyl- ligand nonporous polymer-based gel by elution with a step gradient of sodium perchlorate concentration, and cholesterol concentrations of fractionated lipoproteins were measured by a post-column reaction with a reagent containing cholesterol esterase and cholesterol oxidase. HDL, LDL, IDL, VLDL and chylomicron were separated in this order within 25 minutes, and cholesterol levels of these lipoproteins were measured enzymatically.

### Statistics

For the comparison study, linear regression analyses between the two methods (RLP-C assay and RemL-C assay) were analyzed by Pearson product-moment correlation test. The correlations of RLP-C or RemL-C values to IDL cholesterol levels, measured by the HPLC method, were also analyzed. In addition, Bland & Altman method was used to perform the concordance analysis between RLP-C values and RemL-C values. This analysis graph displays a scatter diagram of the differences plotted against the averages of the two measurements. The upper and lower limits of concordance were calculated as the mean difference plus/minus 1.96 times standard deviation (SD). A value of p < 0.05 was considered statistically significant.

## Results and Discussion

**Figure 1:**
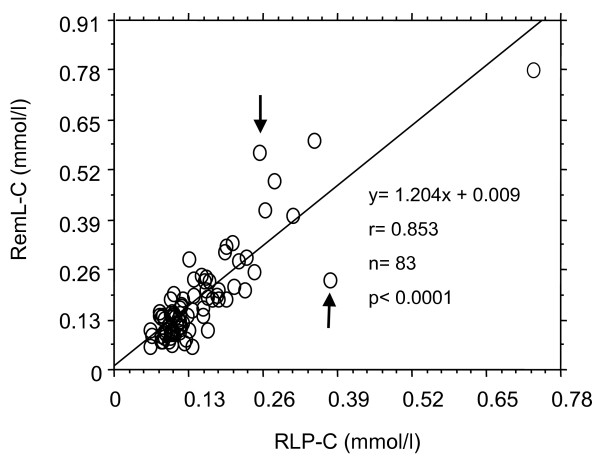
**Correlation between RemL-C and RLP-C values**. Arrows indicate value dissociations of remnant lipoprotein cholesterol level between RemL-C assay and RLP-C assay.

**Figure 2:**
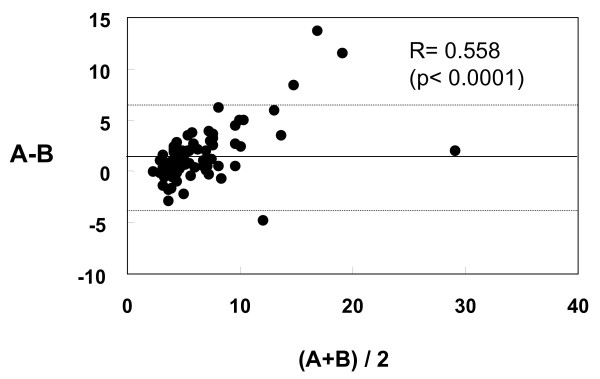
**Bland & Altman analysis for data validation between RemL-C and RLP-C**. A and B indicate RemL-C and RLP-C value, respectively. Namely, X-axis shows RemL-C value + RLP-C value)/2, and Y-axis means RemL-C value – RLP-C value. The Bland & Altman analysis between RemL-C and RLP-C showed a significant proportional error (r = 0.558, p < 0.0001), implicating that RemL-C values are likely to be significantly higher than RLP-C values.

First, we evaluated the correlation of remnant lipoprotein cholesterol concentrations between RemL-C and RLP-C assay. A positive correlation (r = 0.853, 95%CI 0.781–0.903, p < 0.0001) was significantly found between the two methods as shown in Figure 1, while value dissociations between the two methods were also observed in several samples. As shown in Figure 2, the Bland & Altman analysis between RemL-C and RLP-C showed a significant mean difference (1.48 ± 2.67, 95%CI 0.9–2.07) and a significant proportional error (r = 0.558, p < 0.0001). Namely, RemL-C values are likely to be significantly higher than RLP-C values, observed particularly in samples with high levels of remnant lipoproteins.

**Figure 3:**
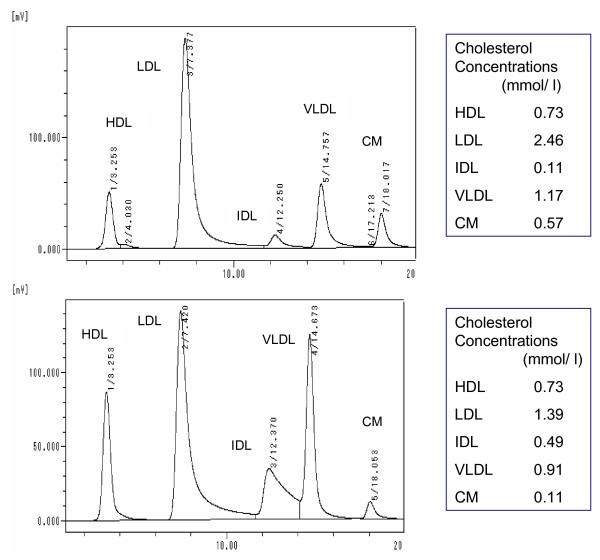
**HPLC chromatograms of lipoproteins from the discrepant samples**. Upper and lower panels indicate the HPLC lipoprotein chromatograms of the samples with RemL-C value < RLP-C value and with RemL-C value > RLP-C value, respectively. CM means chylomicron.

Next, we investigated the lipoprotein profiles of these data-mismatched samples by using the HPLC method to find out the lipoprotein details with regard to the data discrepancies between the two methods. Serum samples with RemL-C value < RLP-C value showed high concentrations of chylomicron cholesterol, while samples with RemL-C value > RLP-C value showed high concentrations of IDL-cholesterol, as shown in Figure 3 which demonstrates the representative HPLC chromatograms, indicated by arrows in Figure 1, in such dissociated samples. In all the samples (n = 83), RemL-C values (r = 0.339, 95%CI 0.152–0.903, p = 0.0005) significantly correlated with IDL-cholesterol concentrations but RLP-C values did not (r = 0.17, 95%CI -0.047–0.372, p = 0.1237). Therefore, RemL-C is likely to be more closely associated with IDL-cholesterol than RLP-C. However, RLP-C might be associated with large-sized TG-rich lipoproteins such as chylomicron and chylomicron remnants in contrast to the close association of RemL-C to IDL. HPLC lipoprotein analysis data showed significant correlations of RemL-C and RLP-C to VLDL-cholesterol [r = 0.538 (p < 0.0001) and r = 0.382 (p = 0.0003), respectively] and chylomicron-cholesterol [r = 0.501 (p < 0.0001) and r = 0.383 (p = 0.0003), respectively). Thus, such similar trends in correlations of remnant lipoprotein cholesterol to VLDL and chylomicron were found in the both methods.

The present study was performed to compare the characteristics of the RemL-C (homogenous assay) and RLP-C (immunoseparation assay) for the measurement of cholesterol levels of TG-rich remnant lipoproteins. The significant correlations between the two methods (RemL-C and RLP-C) have been reported in general samples, while discrepancies between them were also observed particularly in cases with increased IDL, qualitatively estimated, in patients with diabetes [12]. Previous papers have reported that RLP particles are little resemble to classical VLDL remnants, which is smaller than nascent VLDL particles and not enriched in apolipoprotein E relative to apolipoprotein Cs. In addition, Leary et al. reported a high association between buoyant VLDL-cholesterol and RLP-C values but a modest association between IDL-cholesterol and RLP-C [10]. In the present study, the HPLC lipoprotein analysis also showed no significant association between RLP-C and IDL-cholesterol. The quantitative analysis by the HPLC assay shows that RLP-C values are likely to poorly reflect smaller-sized TG-rich lipoprotein remnants such as IDL particles.

In the RemL-C method, POE-POB, a special surfactant can increase the affinity to IDL particles [12]. In the presents study, RemL-C was significantly associated with IDL but RLP-C was not. It may be clinically important to know the association of remnant lipoprotein cholesterol (RemL-C) to IDL-cholesterol, because increased IDL-cholesterol levels have been found to be associated with the incidence of cardiovascular diseases in epidemiological studies [16-18]. However, the RemL-C assay is prone to overestimate remnant lipoprotein cholesterol levels in comparison with RLP-C assay as shown in Figure 2. In addition to increased affinity to IDL by POE-POB, the affinity to chylomicron remnant is also enhanced by phospholipase D. For that reason, RemL-C values may tend to be higher than RLP-C values.

## Conclusion

In conclusion, these results suggest that there is generally a significant correlation between RemL-C, a novel homogenous assay and RLP-C, a conventional immunoseparation method. However, RemL-C assay is likely to reflect IDL more closely than RLP-C. Because of homogenous simple method as opposed to RLP-C, RemL-C assay may be useful for screening individuals with increased IDL, potent atherogenic remnant lipoprotein although the modest overestimation of remnant lipoprotein cholesterol levels by RemL-C assay remains to be resolved.

## List of abbreviations

CI: confidence interval; HDL: high density lipoprotein; HPLC: high performance liquid chromatography; IDL: intermediate density lipoprotein; LDL: low density lipoprotein; POE-POB: polyoxyethylene-polyoxybutylene; RemL-C: remnant lipoprotein cholesterol; RLP-C: remnant-like particle-cholesterol; TG: triglyceride; VLDL: very low density lipoprotein.

## Competing interests

The authors declare that they have no competing interests.

## Authors' contributions

HY made substantial contributions to the study conception and design, data acquisition, data analysis and interpretation, and paper writing. HK carried out data acquisition, data analysis and interpretation. YH carried out data acquisition, data analysis and interpretation. YO carried out data acquisition and data analysis. KIk contributed to the study design and data acquisition. IA carried out data acquisition. SS carried out data acquisition. KD carried out data acquisition and data analysis. KIt carried out data acquisition. HY carried out data acquisition and data analysis. NT contributed to the study design and data acquisition. All authors read and approved the final manuscript.
